# High Dialysate Calcium Concentration is Associated with Worsening Left Ventricular Function

**DOI:** 10.1038/s41598-019-38887-y

**Published:** 2019-02-20

**Authors:** V. B. Silva, T. A. Macedo, T. M. S. Braga, B. C. Silva, F. G. Graciolli, W. V. Dominguez, L. F. Drager, R. M. Moysés, R. M. Elias

**Affiliations:** 1 Nephrology Service, Hospital das Clinicas HCFMUSP, Universidade de São Paulo, São Paulo, Brazil; 2 Heart Institute (InCor), Universidade de São Paulo, São Paulo, Brazil; 3 Universidade Nove de Julho (UNINOVE), São Paulo, Brazil

## Abstract

Dialysate calcium concentration (d[Ca]) might have a cardiovascular impact in patients on haemodialysis (HD) since a higher d[Ca] determines better hemodynamic tolerability. We have assessed the influence of d[Ca] on global longitudinal strain (GLS) by two-dimensional echocardiography using speckle-tracking imaging before and in the last hour of HD. This is an observational crossover study using d[Ca] 1.75 mmol/L and 1.25 mmol/L. Ultrafiltration was the same between interventions; patients aged 44 ± 13 years (N = 19). The 1.75 mmol/L d[Ca] was associated with lighter drop of blood pressure. Post HD serum total calcium was higher with d[Ca] 1.75 than with 1.25 mmol/L (11.5 ± 0.8 vs. 9.1 ± 0.5 mg/dL, respectively, p < 0.01). In almost all segments strain values were significantly worse in the peak HD with 1.75 mmol/L d[Ca] than with 1.25 mmol/L d[Ca]. GLS decreased from −19.8 ± 3.7% at baseline to −17.3 ± 2.9% and −16.1 ± 2.6% with 1.25 d[Ca] and 1.75 d[Ca] mmol/L, respectively (p < 0.05 for both d[Ca] vs. baseline and 1.25 d[Ca] vs. 1.75 d[Ca] mmol/L). Factors associated with a worse GLS included transferrin, C-reactive protein, weight lost, and post dialysis serum total calcium. We concluded that d[Ca] of 1.75 mmol/L was associated with higher post dialysis serum calcium, which contributed to a worse ventricular performance. Whether this finding would lead to myocardial stunning needs further investigation.

## Introduction

Patients with chronic kidney disease (CKD) on dialysis have a high cardiovascular risk and mortality rates^1^ that are not fully explained by classical risk factors such as hypertension, left ventricular (LV) hypertrophy, and diabetes^2^. Haemodialysis (HD) treatment might drive the high cardiovascular mortality, marked by sudden cardiac death and heart failure^3^ and, indeed, HD per se, is a cardiovascular stressor that can precipitate recurrent myocardial ischemia (HD-induced myocardial stunning), leading to the development of LV dysfunction, myocardial hibernation and fibrosis, culminating in heart failure^4^.

Two-dimensional speckle tracking imaging (STI) with 2D strain analysis is a more sensitive method than conventional echocardiography for subtle LV dysfunction assessment^5–7^. This technique is also considered a more powerful predictor of mortality in the general population^8^ and among patients on HD^9^. LV global peak systolic longitudinal strain (GLS), obtained from 2D strain analysis, is the ratio of the maximal change in myocardial longitudinal length in systole to the original length. LV myocardium shortens during systole in the longitudinal direction. Accordingly, GLS has a negative value, and a less negative GLS value indicates worse global LV systolic function^6,10^.

HD-induced regional LV dysfunction has been associated with high volumes of ultrafiltration, and it can be alleviated by cooling dialysate^11^ or by more frequent haemodialysis^12^. These results suggest that a better hemodynamic tolerability might potentially avoid regional LV dysfunction. HD can impair diastolic LV function, and studies yield contradictory results on the effect on systolic function^13,14^.

Calcium in cardiomyocytes is the key element of excitation-contraction coupling, so that hypercalcemia impairs the relaxation^15^. However, the influence of dialysate calcium concentration - d[Ca] is still overlooked and based on simple echocardiogram^16,17^. Since there is no consensus on the ideal d[Ca], there is great variability of choice among countries, mostly 1.25 mmol/L in the United States and >1.25 mmol/L in Europe, Australia and Latin America^18^. The d[Ca] might impact the left ventricular behavior during dialysis, as while a higher concentration determines better hemodynamic tolerability^19^, it can also induce a greater impairment on the left ventricular relaxation^16^. In the current study, we conducted a prospective cross-over study to ascertain whether d[Ca] would have an impact on GLS during HD.

## Results

### Baseline characteristics

We initially invited 23 patients to participate, but after 4 exclusions due to poor echocardiogram image quality, 19 patients were included. Clinical and demographic characteristics of the patients are shown in Table 1. Ten patients (52.6%) were considered nourished according to 7-point scale subjective global assessment (SGA), whereas 9 (47.4%) were classified as having mild/moderate undernourishment. Nourished patients presented lower transferrin [157 (132,177) vs. 174 (162, 190), p = 0.024]. Eight patients (42.1%) presented LV hypertrophy and LV ejection fraction (LVEF) were preserved in all patients (66.7 ± 3.7%).

**Table 1:** Baseline characteristics.

Characteristic	
Age, y	44 ± 13
Male gender, n (%)	6 (31)
BMI, kg/m^2^	23.6 ± 4.8
Hemodialysis duration (months)	44 (30–111)
Hypertension, n (%)	16 (84)
Diabetes mellitus n (%)	5 (26)
Previous parathyroidectomy n (%)	1 (5.3)
**Causes of renal disease**, **n (%)**	
Diabetic Nephropathy	5 (26)
Chronic Glomerulonephritis	3 (15)
Nephrosclerosis	5 (26)
Adult Polycystic Kidney Disease	2 (10)
Others	4 (20
**Drugs**, **n (%)**	
ACEI/ARB	7 (36)
CCB	7 (36)
β-Blocker	10 (52)
Erythropoiesis-stimulating agent	15 (79)
Statin	9 (47)
**Echocardiogram**	
Interventricular septum, mm	11.4 ± 1.9
Posterior wall thickness, mm	11.1 ± 1.8
LV mass indexed to BSA, g/m^2^	116 ± 35
**Laboratory analyses**	
Serum albumin, g/dL	4.0 ± 0.3
Ferritin, ng/mL	433 (310–770)
Transferrin, mg/dL	163 ± 21
Troponin, ng/mL	0.04 ± 0.02
C-reactive protein, mg/dL	1.5 (0.9–2.9)
Aldosterone, ng/dL	11.9 (3.6–73.0)
25(OH) vitamin D, ng/mL	39.0 ± 15.9
Parathyroid hormone, pg/mL	388 (195–544)
Alkaline phosphatase, UI/L	81 (65–116)
Hemoglobin, g/dL	11.2 ± 1.3
α-2-Macroglobulin, mg/dL	247 ± 118

Continuous data were tested for normality and summarized using mean ± SD or median (25–75), as appropriate. BMI, body mass index; LVH, left ventricular hypertrophy; ACEI, angiotensin-converting enzyme inhibitor; ARB, angiotensin II receptor blocker; CCB, calcium-channel blocker; LV, left ventricular; BSA, body surface area.

### Effect of d[Ca] on hemodynamic and biochemical parameters

Comparing both HD sessions with d[Ca] 1.25 and 1.75 mmol/L, the ultrafiltration was the same (3063 ± 534 mL), and weight loss was similar (−2.7 ± 0.8 vs. −2.6 ± 0.6 kg, respectively; p = 0.653), as were pre-dialysis weight, systolic, diastolic and mean blood pressure (MBP).

HD promoted several changes in blood pressure and biochemical parameters. The use of d[Ca] 1.75 mmol/L was associated with a slight drop only in systolic blood pressure and with an increase in serum calcium when compared to d[Ca] 1.25 mmol/L (Table 2). Calcium mass transfer was more positive with d[Ca] 1.75 than 1.25 mmol/L [906 (221; 1,907) mg and 165 (−27; 1,006) mg, respectively, p = 0.023].

**Table 2:** Clinical and biochemical parameters pre and post haemodialysis with d[Ca] 1.25 and 1.75 mmol/L.

Variable	d[Ca] 1.25 mmol/L		d[Ca] 1.75 mmol/L	
	Pre HD	Post HD	Pre HD	Post HD
SBP, mmHg	155.2 ± 1.4	120.2 ± 23.3*	153.7 ± 18.3	140.8 ± 24.7*^a^
DBP, mmHg	85.2 ± 13.2	68.3 ± 16.2*	85.4 ± 13.3	85.0 ± 14.5^a^
MBP, mmHg	108.5 ± 13.5	85.6 ± 17.3*	108.2 ± 11.9	103.6 ± 15.9^a^
Serum TCa, mg/dL	8.9 ± 0.7	9.1 ± 0.5	8.9 ± 0.8	11.5 ± 0.8*^a^
Serum iCa, mg/dL	4.8 ± 0.4	4.3 ± 0.2*	4.7 ± 0.4 2	5.5 ± 0.4*^a^
Potassium, mmol/L	5.7 ± 0.6	3.5 ± 0.2*	5.8 ± 0.7	3.8 ± 0.3*
Hemoglobin, g/L	10.9 ± 1.0	12.6 ± 1.3*	11.2 ± 1.3	13.2 ± 1.6*
Magnesium, mEq/L	2.5 ± 0.4	1.9 ± 0.1*	2.5 ± 0.4	1.9 ± 0.1*
Phosphate, mg/dL	4.7 ± 1.0	1.9 ± 0.6*	4.9 ± 0.9	2.4 ± 0.5*

Data were presented using the mean ± SD. SBP, systolic blood pressure; DBP diastolic blood pressure; MBP, mean blood pressure; TCa, total calcium; iCa, ionized calcium.

*p < 0.05 vs. Pre HD; ^a^p < 0.05 vs. d[Ca] 1.25 mmol/L.

### Baseline GLS

Baseline GLS was −19.8 ± 3.7, ranging from −26.1 to −10.4%, and it was correlated with transferrin (r = −0.648, p = 0.007), troponin (r = 0.573, p = 0.050), and aldosterone (r = −0.555, p = 0.017), and there was also a trend towards a correlation with albumin (r = −0.426, p = 0.078) and alpha-2 macroglobulin (r = −0.448, p = 0.062). There was no difference on baseline GLS when comparing well-nourished and mild/moderately undernourished patients (−20.7 ± 3.0 vs. −18.9 ± 4.4, respectively; p = 0.302).

### Segmental longitudinal strain

Segmental longitudinal strain values were significantly worse in the peak of HD with d[Ca] 1.75 mmol/L compared to baseline in almost all segments (Table 3).

**Table 3:** Segmental strain values at baseline and at the peak of haemodialysis using d[Ca] 1.25 and d[Ca] 1.75 mmol/L.

Segment	Baseline	Peak dialysis Ca 1.25 mEq/L	Peak dialysis Ca 1.75 mEq/L	P (Anova)
Basal anterior	−13.4 ± 5.9	−10.3 ± 6.8	−7.3 ± 7.7*	**0.006**
Basal antero-septal	−12.7 ± 3.3	−9.9 ± 5.0*	−11.4 ± 3.3	**0.042**
Basal infero-septal	−17.4 ± 3.9	−14.0 ± 6.1	−13.9 ± 3.6*	**0.033**
Basal inferior	−14.7 ± 4.8	−11.2 ± 5.8	−9.2 ± 8.1*	**0.045**
Basal infero-lateral	−11.2 ± 7.3	−10.0 ± 6.1	−8.2 ± 9.2	0.342
Basal antero-lateral	−14.7 ± 5.1	−11.0 ± 5.1*	−8.0 ± 6.5*	**0.001**
Midanterior	−19.8 ± 5.3	−14.2 ± 9.5	−13.3 ± 9.3*	**0.007**
Midantero-lateral	−17.0 ± 5.0	−13.9 ± 6.6	−15.6 ± 4.2	0.075
Midinfero-septal	−19.2 ± 5.2	−15.8 ± 7.1	−15.6 ± 3.9	0.052
Midinferior	−17.0 ± 3.8	−12.8 ± 7.0	−11.6 ± 6.8*	**0.031**
Midinfero-lateral	−16.0 ± 7.2	−14.7 ± 6.0	−15.2 ± 4.8	0.701
Midantero-septal	−18.3 ± 5.0	−15.4 ± 7.3	−12.6 ± 6.4*	**0.020**
Apical anterior	−25.3 ± 8.7	−23.6 ± 5.3	−18.8 ± 10.0	0.085
Apical septal	−26.5 ± 6.9	−21.9 ± 11.6	−21.1 ± 6.8	0.059
Apical inferior	−26.1 ± 7.6	−20.2 ± 12.5	−20.0 ± 6.8*	**0.047**
Apical lateral	−25.6 ± 5.3	−21.5 ± 10.4	−20.7 ± 5.2	0.087
Apical	−25.8 ± 6.8	−21.0 ± 11.8	−20.1 ± 6.1	0.061

Data were presented using mean ± SD. *p < 0.05 *vs*. baseline.

### GLS at the peak of haemodialysis

**Figure 1:**
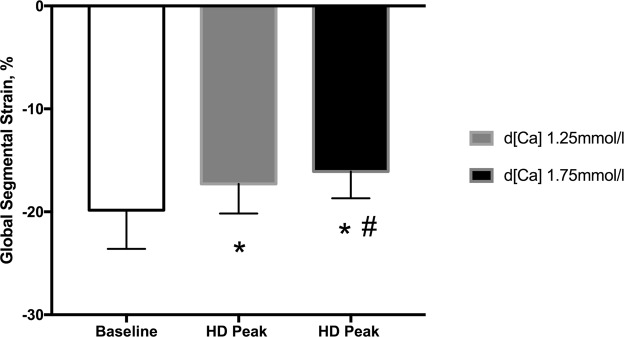
Global Longitudinal Strain (GLS) at baseline and at the peak of HD with d[Ca] 1.25 (gray bar) and 1.75 mmol/L (black bar). *p < 0.05 vs. baseline; ^#^p < 0.05 vs. HD peak d[Ca]1.25 mmol/l.

**Figure 2:**
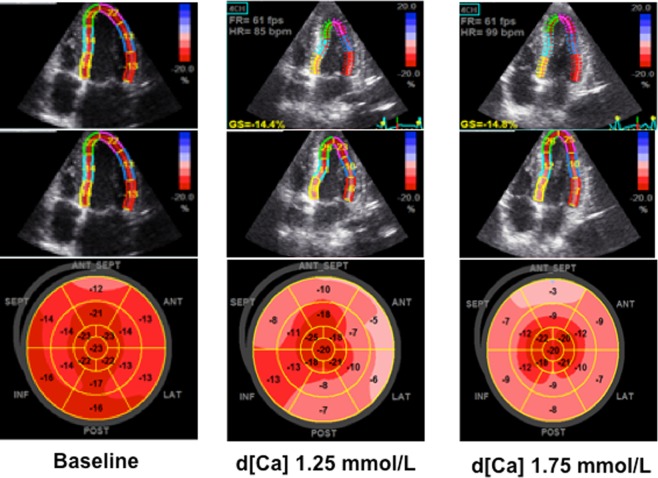
Four-chamber view and peak longitudinal strain values on a Bull’s Eye diagram of all left ventricular segments. The graphical representation of systolic function is arranged in the following order: baseline, d[Ca] 1.25 mmol/L and d[Ca] 1.75 mmol/L. The diagram is obtained as a result of the analysis of basic apical views: four-chamber, two-chamber and left ventricular long axis view. The diagram is presented in the form of a color-coded map for all segments with the values of peak systolic strain of each segment. A lighter color means worse left ventricular dysfunction, which was evident at the peak of haemodialysis, particularly using d[Ca] 1.75 mmol/L.

GLS was worse at the peak of HD compared to baseline (p < 0.001), and it was even worse with d[Ca] of 1.75 than 1.25 mmol/L (−16.1 ± 2.6% vs. −17.3 ± 2.9%, respectively; p < 0.001) (Fig. 1). An example of echocardiogram images illustrating GLS at baseline and at the peak of both HD with d[Ca] 1.25 mmol/L and d[Ca] 1.75 mmol/L is shown in a Bull’s eyes graphical representation in Fig. 2.

GLS at the peak of HD correlated with baseline GLS (r = 0.554, p < 0.001), transferrin (r = −0.599, p < 0.001) and C-reactive protein (r = 0.407, p = 0.012). The correlation with parathyroid hormone (PTH) was non significant (r = 0.304, p = 0.064).

### Mean GLS variation from baseline to the peak of HD (GLS-change)

Regardless of d[Ca], while analyzing the entire population, GLS-change was 3.15 (1.35, 5.38), ranging from −3.80 to 9.50. GLS-change correlated with baseline GLS (r = −0.635, p < 0.001), aldosterone (r = 0.503, p = 0.002), weight loss during dialysis (r = −0.524, p < 0.01), and ultrafiltration (r = 0.553, p < 0.001). There was a trend toward a correlation with albumin (r = 0.309, p = 0.063) and troponin (r = −0.307, p = 0.092). Neither blood pressure nor calcium balance correlated with GLS-change.

### High PTH subgroup analysis

Patients with a PTH higher than 300 pg/mL when compared to the remaining group presented worse GLS at the peak of dialysis (above the median), regardless the d[Ca] (78.9% vs. 21.1% with PTH > 300 mpg/mL and ≤300 pg/mL, respectively, p = 0.009), which represents a 6.5-fold higher risk. Multivariate linear regression analysis showed that GLS at the peak of HD was dependent on transferrin, C-reactive protein and higher post dialysis serum calcium that together explained 66.7% of the variability in GLS (Table 4). GLS-change was worse among patients with PTH > 300 pg/mL than in those with PTH ≤ 300 pg/mL [4.35 (2.5, 6.1) vs. 2.15 (−0.45, 3.18) pg/mL, respectively; p = 0.019], regardless the d[Ca].

**Table 4:** Multiple linear regression of independent factors associated with Global Longitudinal Strain (GLS) at the peak of haemodialysis.

Variable	Beta coefficient	95% CI Lower/Upper	p
Transferrin, mg/dL	−0.055	−0.089/−0.023	**0.001**
C-reactive protein, mg/L	0.274	0.236/0.426	**0.015**
Post dialysis serum calcium, mg/dL	0.477	0.285/0.882	**0.041**
Baseline GLS	0.207	−0.043/0.507	0.110
Weight loss during dialysis, kg	−0.590	−1.117/1.183	0.308

Total model adjusted R^2^ = 0.667, p = 0.001; CI, confidence interval.

## Discussion

Our study provides new insights into the pathogenesis of LV dysfunction on conventional HD, showing an association with the dialysate calcium concentration. We have demonstrated that, despite the relatively better hemodynamic stability with d[Ca] 1.75 mmol/L, this dialysis bath was associated with worsening GLS at the peak of HD, which seems to be related to a higher serum calcium after the procedure. In addition, our data suggest that levels of PTH > 300 pg/mL might also represent a high risk of HD-induced myocardial ischemia, suggesting this hormone might increase the risk of myocardial stunning, although whether this is an independent risk factor warrants further investigation.

We have included a relatively young population, with preserved ventricular function, receiving adequate dose of dialysis, on regular thrice-weekly hemodialysis. Even in this clinical scenario, we observed a compromised ventricular function during hemodialysis, particularly when using d[Ca] of 1.75 mmmol/L, independent of ultrafiltration and blood pressure dropping. Our results, however, should be interpreted with caution since we studied a small sample size.

GLS is a more sensitive predictor for all-cause mortality than LVEF in the general population^20^. Liu and cols. have recently showed that a less negative GLS (defined as GLS ≥ −15%) predicted all-cause and cardiovascular (CV) mortality in HD patients with preserved LVEF^21^. Another study that enrolled 183 patients who were followed for 7.8 ± 4.4 years demonstrated that worsening GLS was independently associated with a higher all-cause and CV mortality in patients with stage 4, 5 CKD who were on HD^22^. A recent study has used magnetic resonance imaging to examine acute effects of standard HD versus hemodiafiltration in stable patients and, similarly to our findings, showed that all patients experienced some degree of segmental left ventricular dysfunction^23^. Interestingly, in the mentioned study by Buchanan C. *et al*.^23^ the d[Ca] was 1.5 mmol/L, which was not tested in the current study.

The association between d[Ca] and mortality in HD patients is still debatable. Data of The Dialysis Outcome Practice Pattern Study (DOPPS) have shown that high serum calcium was associated with increased all-cause mortality in HD patients^24^. Similarly, a recent observational study has demonstrated that HD with d[Ca] 1.75 mmol/L was associated with increased all-cause mortality compared with HD with d[Ca] 1.25 mmol/L^25^, which is not a unanimous finding^26^, since authors have demonstrated that a very low d[Ca] (<1.25 mmol/L) can be associated with an increased risk of sudden cardiac arrest, instead^27^.

The positive calcium balance generated from a high d[Ca] might contribute to increased mortality in HD patients, since a repeated exposure to calcium load has been associated with arterial calcification and stiffness^28^, which are associated with an increased risk of mortality in these patients^29^. The ideal dialysate calcium concentration is probably still unknown. However, data from the literature show that high d[Ca] has also been associated with high sympathetic stimulus during HD^30^, which might impact long-term mortality^31^. Kidney Disease Outcomes Quality Initiative (K/DOQI) guidelines have recommend a prescription of low d[Ca] to maintain neutral calcium balance and reduce vascular calcification^32^. However, reducing calcium load during HD may lead to a decreased hemodynamic stability through changes in systemic vascular resistance and/or cardiac output^33^. In the current study, we have tested the hypothesis that the d[Ca] would directly influence LV dysfunction because higher concentrations lead to a better hemodynamic tolerability^19,34^ and because it is related to a higher sympathetic activity^30^, whereas lower concentrations do the opposite. We found that 1.75 mmol/L d[Ca] was associated with a higher post dialysis serum calcium and worse GLS. We can speculate that since blood flow into the coronary arteries is greatest during ventricular diastole, higher d[Ca] could caused an impairment of coronary flow reserve. Ineffective vasoregulation predisposes the body to myocardial ischemia, which is already compromised in patients on dialysis^35^. The fact we found the association between a worse GLS with post dialysis serum calcium but not with calcium balance is still debatable. Our results suggest that, at least for the myocardial performance, serum calcium has a stronger impact than the calcium balance. Moreover, a positive calcium balance does not necessarily mean an increase in serum calcium, as the skeleton might act as a continuous buffering, mainly in those patients with higher serum PTH^36^. Our study emphasizes the need to elucidate the independent role of serum calcium and d[Ca] on the ventricular dysfunction and whether this contributes to myocardial stunning and increasing mortality risk in dialysis patients.

Elevated troponin concentration is associated with high mortality in dialysis patients^20,37,38^, even in patients with preserved LVEF^21^. High troponin levels are clearly associated with cardiac structural and functional damage, as LV hypertrophy, LV dilation, and systolic and diastolic dysfunction^39^. Our results showed an association between less negative baseline GLS and increased troponin concentration, which were consistent with a previous study^21^, and may reflect the presence of subtle LV dysfunction, detected by GLS, and subclinical myocardial injury resulting in the increase of troponin. Dialysis-induced myocardial stunning probably plays a role in this process, leading to systolic dysfunction, as demonstrated by McIntyre and cols^4^, showing a less negative GLS. Furthermore, it has been associated with high troponin levels^40^.

The correlations we found between GLS at the peak of HD and inflammation was already described^12^. We have confirmed this finding by showing a correlation between GLS at the peak of HD and C-reactive protein and extending the association with another inflammatory marker, the alpha-2 macroglobulin. Nutritional status has been associated with the risk of HD-induced myocardial stunning^41^. We found a correlation between GLS and serum transferrin, a sensitive marker for nutritional status and marker of protein-energy wasting^42^. Transferrin has a shorter half-life compared with albumin, which gives it a theoretical advantage as a nutrition marker. Despite this finding, we did not confirm a correlation between GLS and other markers of nutritional status such as albumin. In addition, there was no difference on baseline GLS when comparing well-nourished and mild/moderately undernourished patients.

PTH, a calcium regulator hormone, is also a depressive myocardial factor. We found that PTH, indeed, had an influence on the delta of GLS, which was worse in patients with levels of PTH higher than 300 pg/mL. Uremia, PTH and phosphate were already implicated in the cardiac remodeling process in CKD^43^. Based on this knowledge, we can postulate that PTH might have a direct effect on the myocardial response during HD. On the other hand, indirect effects of PTH can be noted since parathyroidectomy status^34^ and levels of PTH^36^ can interfere with hemodynamic changes and calcium balance during a conventional HD.

The present study has a unique strength in showing, for the first time, an influence of d[Ca] on HD-induced ventricular dysfunction, measured by GLS. In addition, this study has been conducted keeping constant ultrafiltration rate and a low temperature, which allowed the study of d[Ca] as an independent risk factor, and the study was single-blinded and prospective. Despite these strengths, the results of our study need to be interpreted in light of its limitations. There was only a single HD studied, the carry-on effect between the interventions could not be completely discarded, the sample size was relatively small, and we cannot guarantee that body temperature was stable in all patients during the 2 interventions. Patients included in the study are relatively young, with reasonable cardiac function, and therefore our results should be confirmed in larger population before can be widespread. In addition, we cannot exclude the possibility that our findings are confounded by factors that we could not ascertain, such as the hydration status.

In summary, we have demonstrated that a 1.75 mmol d[Ca] might cause a worsening of GLS when compared to 1.25 mmol d[Ca], even using a cool dialysate. Our study is hypothesis generating, and more studies should be performed, as the exact mechanism remains to be elucidated. The clinical impact of using long-term high d[Ca] on ventricular dysfunction and whether this is related to myocardial stunning and long-term mortality in this population is still undefined.

## Methods

### Study Population

This was a single-center, single blind, and crossover study. The echocardiogram and the cardiac imaging analyses were blinded to patient details and treatment group allocations. Patients on thrice weekly conventional HD were enrolled after a recruitment period from July 2015 to April 2016. During the study no patient was receiving calcimimetic and one patient was submitted to parathyroidectomy 3 years previous to the study entry. The inclusion criteria were patients >18 years old who were on conventional HD for at least 6 months. Exclusion criteria were hospitalization in the last 6 months due to an active episode of decompensated heart failure or acute coronary syndrome, atrial fibrillation or another arrhythmia and poor echocardiographic image quality. The Local Institution Review Board at the Hospital da Clinicas da Universidade de São Paulo (Cappesq# 30284714.0.0000.0068) has approved the study protocol, which was conducted in accordance with the Declaration of Helsinki. All participants provided written informed consent to participate in the study. The protocol was registered at ClinicalTrials.gov (NCT02545426).

### Study Procedures

#### Dialysis

The treatment duration was 4 hours, with a dialysate-flow rate of 800 mL/min and a blood-flow rate of 300 mL/min. All patients received unfractionated heparin as required and used high-flux polysulfone dialyzers (Fresenius® FX60; Fresenius Germany). The dialysate temperature was set at constant value of 35.5 °C. Twelve patients (63%) used arteriovenous fistulae as vascular access, and eight had a catheter. The dialysis prescription was adjusted to achieve a urea reduction ratio of 65% and a single pool Kt/V of 1.2 as described by Daugirdas and colleagues^44^. The ultrafiltration was set exactly the same in the two arms of the study for the same patient, and therefore was similar between interventions (3063 ± 534 mL vs. 3063 ± 534 mL, p = 1).

### Echocardiographic Measurements

Two-dimensional echocardiography was performed in the left lateral decubitus position before dialysis and at peak dialysis in both situations (d[Ca] 1.25 and 1.75 mmol/L). A well-trained cardiologist obtained the following echocardiography images: the standard apical 2-, 3- and 4-chamber views before HD and at peak HD (60 minutes before the end of dialysis treatment). The images were obtained (1.5–3.6 MHz 3 S probe, Vivid I; GE Medical Systems, Sonigen, Germany) and were saved for subsequent analysis using a digitizing program (EchoPAC PC version 110.1.3, GE HealthCare, Tirat Carmel, Israel) by a physician totally blinded to the intervention and clinical data. This program uses speckle-tracking imaging (STI) to measure the myocardial strain. The region of interest was traced for each image at the end-systolic frame. Segmental and global values of left ventricular longitudinal myocardium strain were calculated. The operator manually adjusted segments that failed to track. GLS was calculated as the mean strain of all segments. The speckle patterns on a frame-by-frame basis were tracked using the EchoPAC tracking algorithm. Three consecutive heartbeats were analyzed for each image, and the peak strain was measured. A detailed description of STI analysis has been previously described^45^. The echocardiograms were evaluated according to the recommendations suggested by the American Society of Echocardiography^46^. The LVEF was calculated using Simpson’s biplane method. LV mass index was determined as the ratio of left ventricular mass to body surface area.

### Laboratory Measurements

Blood samples were collected for biochemical analysis pre- and immediately post dialysis in the two interventions. All biochemical analyses were done according to the manufacturer’s instructions and usual techniques. Parathyroid hormone (PTH) was measured by chemiluminescence immunoassay (reference range = 11–65 pg/mL; Roche immunoassay analyzer, Roche Diagnostics, Germany). We have a performed a subanalysis of patients with PTH higher than 300 pg/ml, defined as patients with hyperparathyroidism since it was already demonstrated that these patients might have a distinct calcium balance when exposed to the same d[Ca]^36^. Troponin was measured by third-generation electrochemiluminescence assay (reference range = < 0.03 ng/mL; Roche Diagnostics). α-2-Macroglobulin was measured using a Multiplex Milliplex map kit – Human CVD Panel 3 (Acute Phase) – HCVD3MAG-67K (EMD Millipore Corporation, MA, USA®) assay.

### Calcium Balance

Dialysate samples were collected from fresh dialysate and from a homogenous sample of spent dialysate collected during the 4-hour HD procedure to determine mass transfer of calcium. This procedure has been validated in the literature^47,48^.

### Nutritional evaluation

Body mass index was calculated using the weight in kilograms divided by the square of the height in meters. Nutritional status was evaluated by the same observer, using the SGA classification technique as previously described^49^. Briefly, the SGA classification technique used historical data gathered from the patient on weight change, altered dietary intake, gastrointestinal symptoms influencing oral intake/absorption, and a physical examination. Patients were classified as well nourished, mild/moderately undernourished or severely undernourished.

### Exposure

All patients were under regular HD with a d[Ca] of 1.75 mmol/L when were assigned to a random mid-week HD, with high (1.75 mmol/L) or low (1.25 mmol/L) d[Ca] and vive versa, in the subsequent week. The intervention was applied in only one session and patients returned to d[Ca] of 1.75 mmol/L afterward. Ultrafiltration was equal in both study arms.

### Statistical analysis

The results are presented as the mean ± SD or median and (25–75) quartiles depending on the normality of the data. Comparisons between d[Ca] 1.25 and d[Ca] 1.75 mmol/L] were done using Student’s t test or Mann-Whitney test according to the Gaussian distribution. The correlation coefficients were Pearson or Spearman, depending on the normality of the data. Linear multiple regression analysis was performed with GLS at the peak of dialysis as a dependent variable and with the independent variables selected from the univariate analysis (p < 0.05). Due the lack of information about the influence of d[Ca] on GLS, sample size was calculated based on the data obtained with the first 15 patients included (mean difference in GLS between d[Ca] 1.25 and d[Ca] 1.75 mmol/L was 1.2 ± 1.7%). The required sample size to reach 5% of alpha error and 80% of power expected was 18 patients, in a paired design study. A p value < 0.05 was considered significant. Analyses were performed with the use of SPSS 21.0 (SPSS Inc., Chicago, Ill., USA) and GraphPad Prism® software version 7.0 (GraphPad Software, Inc., Calif., USA).
